# Atypical Forms of Congenital Hyperinsulinism in Infancy Are Associated With Mosaic Patterns of Immature Islet Cells

**DOI:** 10.1210/jc.2017-00158

**Published:** 2017-06-09

**Authors:** Bing Han, Zainab Mohamed, Maria Salomon Estebanez, Ross J. Craigie, Melanie Newbould, Edmund Cheesman, Raja Padidela, Mars Skae, Matthew Johnson, Sarah Flanagan, Sian Ellard, Karen E. Cosgrove, Indraneel Banerjee, Mark J. Dunne

**Affiliations:** 1 Faculty of Biology, Medicine & Health, The University of Manchester, Manchester M13 9PT, United Kingdom; 2 Paediatric Endocrinology, The University of Manchester, Manchester M13 9PT, United Kingdom; 3 Paediatric Surgery, Central Manchester University Hospitals NHS Foundation Trust (CMFT) and The University of Manchester, Manchester M13 9PT, United Kingdom; 4 Paediatric Histopathology, Central Manchester University Hospitals NHS Foundation Trust (CMFT) and The University of Manchester, Manchester M13 9PT, United Kingdom; 5 Molecular Genetics, Royal Devon and Exeter NHS Foundation Trust, University of Exeter Medical School, Royal Devon and Exeter Hospital, Exeter EX2 5DW, United Kingdom

## Abstract

**Objectives:**

We aimed to characterize mosaic populations of pancreatic islet cells from patients with atypical congenital hyperinsulinism in infancy (CHI-A) and the expression profile of NKX2.2, a key transcription factor expressed in *β*-cells but suppressed in *δ*-cells in the mature pancreas.

**Patients/Methods:**

Tissue was isolated from three patients with CHI-A following subtotal pancreatectomy. CHI-A was diagnosed on the basis of islet mosaicism and the absence of histopathological hallmarks of focal and diffuse CHI (CHI-D). Immunohistochemistry was used to identify and quantify the proportions of insulin-secreting *β*-cells and somatostatin-secreting *δ*-cells in atypical islets, and results were compared with CHI-D (n = 3) and age-matched control tissues (n = 3).

**Results:**

In CHI-A tissue, islets had a heterogeneous profile. In resting/quiescent islets, identified by a condensed cytoplasm and nuclear crowding, *β*-cells were reduced to <50% of the total cell numbers in n = 65/70 islets, whereas *δ*-cell numbers were increased with 85% of islets (n = 49/57) containing >20% *δ*-cells. In comparison, all islets in control tissue (n = 72) and 99% of CHI-D islets (n = 72) were composed of >50% *β*-cells, and >20% *δ*-cells were found only in 12% of CHI-D (n = 8/66) and 5% of control islets (n = 3/60). Active islets in CHI-A tissue contained proportions of *β*-cells and *δ*-cells similar to those of control and CHI-D islets. Finally, when compared with active islets, quiescent islets had a twofold higher prevalence of somatostatin/NKX2.2^+^ coexpressed cells.

**Conclusions:**

Marked increases in NKX2.2 expression combined with increased numbers of *δ*-cells strongly imply that an immature *δ*-cell profile contributed to the pathobiology of CHI-A.

## Teaser

Atypical forms of congenital hyperinsulinism in infancy were found to be associated with a mosaic profile of immature islet cells. This was directly related to the altered expression of NKX2.2.

Congenital hyperinsulinism in infancy (CHI) is a complex clinical condition associated with inappropriate insulin release from pancreatic *β*-cells, leading to profound hypoglycemia in newborns and early infancy. CHI and the associated hypoglycemia can potentially result in brain damage, with long-term neuronal disabilities occurring in more than one-third of cases (1, 2). Several genetic mutations have been identified as the causes of CHI, which can affect all cells of the islets of Langerhans [diffuse CHI (CHI-D)] or can be localized to a focal lesion of proliferating *β*-cells (focal CHI) (1, 2). Mutations in *ABCC8* (encoding SUR1) and *KCNJ11* (encoding Kir6.2) are associated with the majority of severe and persistent cases of disease (3, 4). Rarer forms of CHI have been described as caused by defects in *HADH* (encoding short-chain L-3-hydroxyacyl-CoA dehydrogenase), *GLUD1* (encoding glutamate dehydrogenase), *GCK* (encoding glucokinase), *HNF4A* (encoding hepatocyte nuclear factor 4*α*), *HNF1A* (encoding hepatocyte nuclear factor 1*α*), *SLC16A1* (encoding monocarboxylate transporter, MCT1), *UCP2* (encoding mitochondrial uncoupling protein 2), *HK1* (hexokinase 1), *PGM1* (phosphoglucomutase 1), and *CACNA1D* (l-type calcium channel *α*-subunit) (1–7). However, there remains a high proportion of patients with persistent CHI [*e.g.,* >60% of CHI patients in Australia (8) and China (9) who have an unknown genetic cause of disease]. Among this cohort are patients with CHI who have been designated with atypical disease (CHI-A) (10, 11).

Apart from an unknown genetic cause, CHI-A has other identifying features that characterize affected patients. CHI-A is associated with late presentation of symptoms, has a declining sensitivity to medications such as diazoxide and somatostatin receptor agonists, and cannot be easily detected by ^18^F-dihydroxyphenylalanine positron emission tomography-computed tomography because it has the same appearance as CHI-D (10–12). In the face of declining responses to medication, surgical removal of the pancreas is required to alleviate hypoglycemia, allowing a definition of CHI-A to be made from a histopathological perspective. This diagnosis relies upon exclusion of focal islet cell hyperplasia (focal CHI) and islet cell nucleomegaly (CHI-D) (13, 14) and the identification of heterogeneous populations of islets, which appear to be resting or quiescent and localized to particular domains/lobes of the pancreas (10, 11, 15). These subjective comparisons of characteristic mosaic abnormalities require access to major amounts of postoperative tissue, and in the absence of other defining histopathological hallmarks, the pancreas of CHI-A patients can be troublesome to define (10, 11). The pathogenesis of the heterogeneous populations of islets in certain localizations in the pancreas remains undetermined but may be developmental in origin.

In patients with CHI-D, we have recently observed alterations in the ontogenic profile of the islets, suggesting that the tissue is closely aligned with a developmentally naive (fetal-like) pancreas rather than age-matched control tissue (16). This was defined as the inappropriate expression of NKX2.2 (Nirenberg and Kim 2 homeobox 2) in islet cells. NKX2.2 is a transcription factor that is important for insulin-secreting *β*-cell identity (17) and normally is suppressed in somatostatin-secreting *δ*-cells after birth (18). However, in islets obtained from CHI-D pancreata, there was a more than threefold increase in the coexpression of NKX2.2 with somatostatin in *δ*-cells (16). These data imply an increase in both insulin-secreting and insulin-suppressing cellular capacity within the islets. Because it is not known whether a similar developmental abnormality is present in CHI-A that may be linked to the mosaic patterns of active and quiescent tissue, we examined whether similar alterations in the developmental profile of CHI islets also occur in atypical disease.

## Materials and Methods

### Patient details

In our center, we identified three patients with CHI-A from a cohort of 42 patients who underwent surgery for CHI. Tissue samples were obtained with ethical permission and informed consent from each patient with CHI-A after subtotal pancreatectomy for sustained hypoglycemia. Two patients were male (birth weights, 3.4 and 3.6 kg), and one patient was female (birth weight, 2.6 kg). All patients exhibited distinct clinical features of CHI-A. These included a late-onset presentation of hypoglycemia at 7 months, 11 months, and 30 months of age and a declining profile in therapeutic responsiveness to diazoxide and octreotide treatment. Although ^18^F-dihydroxyphenylalanine positron emission tomography-computed tomography data were indicative of a diffuse pancreatic abnormality, blood samples from all three patients were negative for known defects in CHI-causing genes. Sequence analysis of the *ABCC8, KCNJ11, HADH,* and *GCK* genes and targeted next-generation sequencing of known CHI genes in blood samples did not identify a pathogenic mutation. All patients underwent a 95% pancreatectomy between 5 and 6 months after presentation of clinical symptoms.

The diagnosis of CHI-A was based on the heterogeneous/mosaic histopathological appearance of tissue involving active or hyperfunctional islets and quiescent islets with a resting appearance (which manifest as nuclear crowding), the heterogeneous expression of islet hexokinase I, and the absence of other defining criteria for focal disease and diffuse disease (diffuse islet hyperplasia, an enrichment of islet cell nuclear hyperplasia) (10, 11, 13–15). Additional genetic analysis of CHI-A was performed by testing DNA samples extracted from pancreatic tissue (following surgery) by examining the coding regions and the exon/intron boundaries of the *ABCC8, KCNJ11, HNF4A, HADH, GCK*, *GLUD1, INSR, SLC16A1, TRMT10A,* and *HNF1A* genes by targeted next-generation sequencing to high depth (mean coverage across genes: 613×) (19). Bespoke analysis for heterozygous and mosaic variants down to a level of 1% did not identify a pathogenic mutation.

### Immunostaining and cell counting

Tissue samples were fixed in 4% paraformaldehyde and embedded in paraffin wax; 5-µm-thick sections were prepared for immunostaining. Immunohistochemistry and dual immunofluorescence labeling were performed on tissue sections as described previously (14, 16, 20), using validated and selective primary antibodies to detect the proteins of interest: insulin (1:1000; Abcam, Cambridge, UK), somatostatin (1:300; Zymed, San Francisco, CA), NKX2.2 (1:75; Developmental Studies Hybridoma Bank, Iowa City, IA), and hexokinase I (1:100; Santa Cruz, Dallas, TX). Images were acquired and digitized by a 20×/0.80 Plan Apo objective using the 3DHistech Pannoramic 250 Flash II slide scanner. Pannoramic Viewer and HistoQuant software packages were used for data analysis and high-content cell counting (3DHISTECH Ltd., Budapest, Hungary). Islets with clear boundaries were selected to quantify the percentage of cells with coexpression of NKX2.2 and somatostatin compared with total islet cell counts. For the quantification of data, islet profiles from CHI-A tissues were directly compared with islets in age-matched tissue from CHI-D (n = 3) as a consequence of *ABCC8* gene defects and from neonatal control tissues (n = 3) as previously described (14, 16, 20).

### Statistical analysis

Where appropriate, data are presented as mean ± standard error. One-way analysis of variance followed by the Tukey *post hoc* test was used to determine significant differences between the means of data sets.

## Results

### Mosaic profiles of islets in the CHI-A pancreas

**Figure 1.**
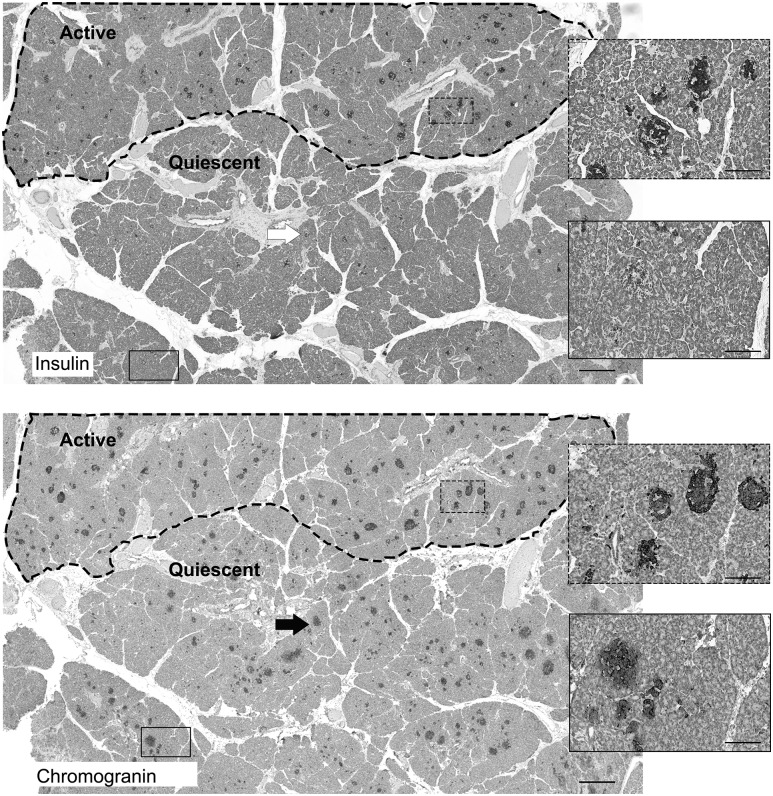
The mosaic arrangement of the endocrine pancreas in CHI-A. Within the dotted region of the pancreas in the upper panel (Active), islets rich with insulin-positive staining are clearly visible. The equivalent islet structures are also visible in the serial section of tissue shown in the lower panel, which was stained for chromogranin A. However, in the area outside the dotted region, insulin expression is weak, suggesting that islets within this domain are limited in *β*-cell numbers and/or are quiescent (Quiescent). Within the quiescent domain, islets are clearly present when stained for the neuroendocrine marker chromogranin A. The arrow and boxed regions show how islets are clearly visible when stained for chromogranin but not insulin. Scale bar = 500 μm; boxed regions = 50 μm.

The typical heterogeneous appearance of islets in CHI-A tissues was revealed by comparing serial sections of tissue stained for either insulin or chromogranin A, a nonspecific marker of neuroendocrine cells (Fig. 1). When insulin expression was used to identify islets through the population of *β*-cells, only islets in the active domains of the pancreas (indicated) were readily seen. In the quiescent part of the pancreas, islets that were poorly visible in the presence of an insulin antibody were clearly labeled in the presence of the chromogranin A antibody. This suggests that islets in the active parts of the tissue contained abundant *β*-cells compared with quiescent islets.

**Figure 2.**
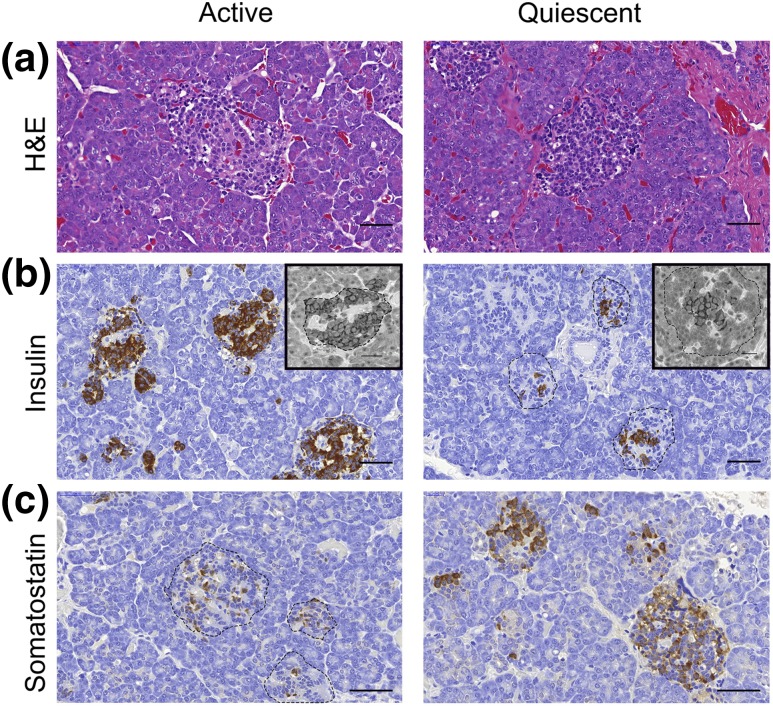
Diverse islet profiles in the pancreas of CHI-A tissues. (a) Within regions that have a normal active appearance, (b) islets are enriched with insulin-expressing *β*-cells and (c) have fewer somatostatin-expressing *δ*-cells. However, within domains that are quiescent, (a) islets have condensed cytoplasm, leading to the appearance of nuclear crowding, (b) fewer *β*-cells, and (c) a higher number of *δ*-cells. Note that the distribution of *β*-cells in quiescent islets is also different and that the cells occupy a central location within the islets. (b) This expression profile was also mirrored when *β*-cells were identified for the expression of proinsulin (inserts). (b, c) Dotted lines have been added to clarify the islet structure. The data are representative of n = 3 CHI-A cases. Scale bar = 50 μm; inserts = 20 μm. H&E, hematoxylin and eosin.

**Figure 3.**
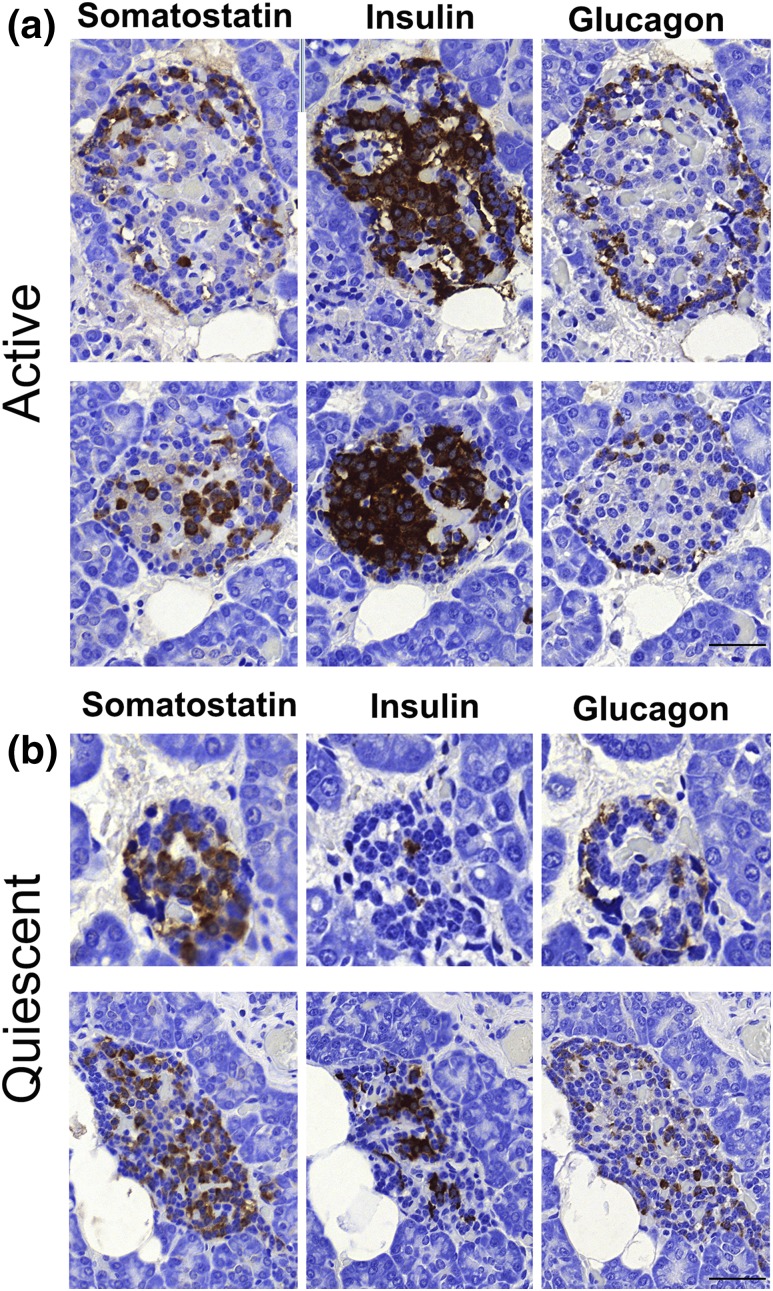
Islet hormone expression in active and quiescent domains of the pancreas of CHI-A tissues. (a) Islets with a normal (active) appearance are enriched with insulin-expressing cells and have lower numbers of somatostatin- and glucagon-expressing cells. (b) In contrast, in the quiescent islets, somatostatin is enriched and the number of *β*-cells is low. Note that there is no difference in the abundance of glucagon-expressing *α*-cells. The data are representative of n = 3 CHI-A cases. Scale bars = 50 μm.

These observations were supported by examining n = 1765 islets in CHI-A tissues (n = 3 cases). Figure 2 summarizes islet cell expression profiles in the active and quiescent domains of the pancreata from CHI-A tissue. In all three cases of CHI-A, quiescent islet cells had the appearance of a resting profile because their cytoplasm was relatively condensed, leading to nuclear crowding within the islet structure [Fig. 2(a)]. In these islets, *β*-cells were limited in number and were located centrally toward the core of the islet [Fig. 2(b)]. Proinsulin with an expression profile that mirrored that of insulin was also detected. In sharp contrast, *δ*-cells were abundant in quiescent islets throughout the tissues and were present in much higher numbers than in active islets [Fig. 2(c)]. There was no alteration in the profiles of glucagon-expressing *α*-cells in the mosaic areas of the pancreata of CHI-A tissues (Fig. 3).

### Enhanced expression of *δ*-cells in quiescent islets

**Figure 4.**
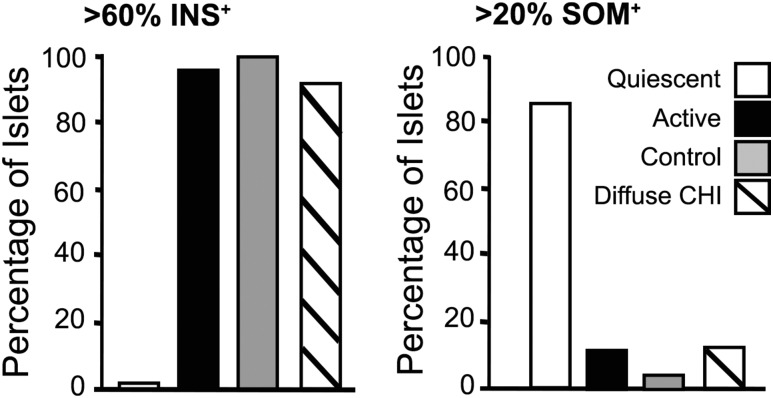
Expression profiles for insulin and somatostatin in mosaic islets. No quiescent islets from CHI-A tissue were composed of >60% *β*-cells (INS^+^) (n = 70 islets). For comparison, >97% of active islets (n = 70) from the same tissues (n = 3 patients), 100% of age-matched control pancreata (n = 70 islets; n = 3 donors), and 93% of CHI-D tissues (n = 72 islets; n = 3 patients) were composed of >60% *β*-cells. In quiescent tissue, 85% of the islets (n = 57) were composed of >20% *δ*-cells compared with 13% of active islets from the same tissue (n = 3 cases; n = 46 islets), 5% of control islets (n = 3 cases; n = 60 islets), and 12% of CHI-D islets (n = 3 patients; n = 66 islets).

To quantify the extent of islet cell heterogeneity with the CHI-A pancreata, we next examined the profiles of selected numbers of islets from the CHI-A tissues and compared these with islets in CHI-D tissues (n = 3 cases) and age-matched control tissues (n = 3 donors). In CHI-D and control tissues, quiescent islets were seen in only <0.75% of the total number of islets quantified (n = 429 and n = 635, respectively). Figure 4 shows that in control tissue, all islets (100%) were composed of 60% or more *β*-cells (n = 70 islets) compared with 93% of islets from CHI-D tissue (n = 72) and 97% of islets (n = 70) examined in active regions of CHI-A tissues. In sharp contrast, no quiescent islet (0%; n = 70) was enriched with >60% *β*-cells, and n = 42/70 islets were found to contain <30% *β*-cells. In the quiescent domains of the CHI-A tissues, 85% of the islets (n = 49/57) were found to contain >20% *δ*-cells, and 54% (n = 31/57) were composed of >50% *δ*-cells. In contrast, only 13% of active islets in CHI-A tissue (n = 6/46), 5% of control islets (n = 3/60), and 12% of CHI-D islets (n = 8/66) were composed of >20% *δ*-cells.

### Altered expression of NKX2.2 in islet *δ*-cells

**Figure 5.**
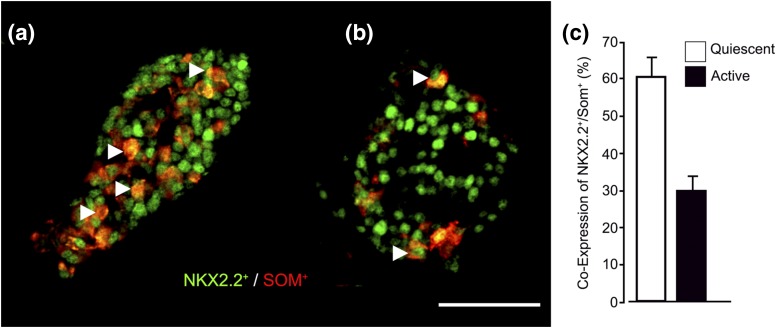
Coexpression of somatostatin and NKX2.2 in CHI-A *δ*-cells. Representative images of the immunofluorescence stains using NKX2.2 (green) and somatostatin (red) colabels show far more cells (a) in quiescent islets than (b) in control islets. Some of the cells that were colabeled with NKX2.2 (NKX2.2^+^) and somatostatin (SOM^+^) are highlighted (arrowheads). (c) Summary of the average proportions of cells coexpressing NKX2.2 and somatostatin in quiescent (n = 10) and active (n = 10) CHI-A islets. Scale bar = 50 μm.

NKX2.2 is a transcription factor of considerable importance for islet cell identity and endocrine cell lineage determination during fetal development. The abnormal fetal-like maintenance of NKX2.2 expression in *δ*-cells has been described in islets of patients with CHI-D (16). In CHI-A tissue, we found that islets composed of high numbers of *δ*-cells were also associated with marked increases in the coexpression of NKX2.2 [Fig. 5(a)]. On average, coexpression of NKX2.2^+^ and somatostatin was twofold higher in quiescent islets (n = 685 cells, 10 islets) than in active islets (n = 204 cells, 10 islets) [Fig. 5(c)]. These data further support the notion that CHI-A has heterogeneous populations of islets and suggest that islets with a quiescent profile are reminiscent of fetal tissue rather than postnatal islets.

## Discussion

CHI is a complex clinical condition primarily linked to 10 known genetic causes in either focal lesions or diffuse islet abnormalities. Most severe cases of CHI are associated with defects in the components of adenosine triphosphate−sensitive K^+^ channels—encoded by *ABCC8* and *KNCJ11*. Loss-of-function mutations have a causal link to uncontrolled insulin release, but CHI-causing mutations are also known to enhance islet cell proliferation in patients with diffuse disease and disruptions in islet cell identity, including nucleomegaly (16, 21, 22). Although the mechanisms associated with alterations in islet cell specification are unknown, they are accompanied by changes in the expression levels of several key transcription factors that are important in the developmental competence of the fetal pancreas. The most relevant interruptions to the normal profile of transcription factor expression involve increases in NKX2.2 expression (16).

During development, NKX2.2 is critical for the differentiation of *β*-cells, with gene defects leading to neonatal diabetes (17, 18, 23, 24). NKX2.2 is also one of the few transcription factors to cause absolute loss of insulin-secreting cells when ablated (18, 24). Furthermore, the loss of NKX2.2 expression results in substantial decreases in the numbers of glucagon-secreting *α*-cells and pancreatic polypeptide-secreting cell numbers but not *δ*-cells numbers, because somatostatin-producing *δ*-cells remain unaffected (24). In the human fetal pancreas, NKX2.2 expression is found in approximately 70% of somatostatin-producing *δ*-cells (17, 24). Although NKX2.2 expression persists in some postnatal *δ*-cells (approximately 25% of cells), the incidence of coexpression with somatostatin is only a fraction of that seen in the fetal pancreas (16, 24), indicating that suppression of NKX2.2 expression is required for *δ*-cell identity and normal function in the postnatal period.

In tissue from patients with CHI-D, we found that NKX2.2 gene expression was markedly elevated compared with expression in age-matched controls, and this was most obvious during the first 6 months after birth (16). Protein expression studies in islets further revealed that NKX2.2 expression persisted in both *β*-cells and somatostatin-producing *δ*-cells in the CHI-D pancreas (16). This particular feature mirrors fetal *δ*-cells and contrasts with the low levels of coexpression observed in postnatal *δ*-cells from control samples, in which approximately 75% of somatostatin-producing cells failed to express NKX2.2 (17).

In contrast to our understanding of focal and diffuse CHI, relatively little is known about other severe forms of CHI. CHI-A is currently used to describe cases of the disease with an unknown genetic basis, a declining sensitivity to therapies, and a histopathology of the pancreas that is characteristically mosaic (10, 11, 15). Without a profile of genetic and histological data sets, the precise incidence of CHI-A is difficult to determine.

In our center, three patients with the criteria of CHI-A were identified from a cohort of 42 patients undergoing pancreatic surgery. Snider *et al.* (25) found that 11 of 282 had a histopathology that could not be described as focal or diffuse, including a subset of patients with localized islet cell nucleomegaly. In this study, we extended the notion that the pathobiology of disease is related to the heterogeneity of islets in CHI-A tissue and critically examined the expression of insulin and somatostatin in different populations of islets. This revealed that quiescent islets are composed of increased numbers of somatostatin-producing cells and greatly diminished numbers of *β*-cells in comparison with control islets (26). Of note, we also found that within these islets >60% of *δ*-cells coexpressed NKX2.2 at proportions similar to those in CHI-D islets and fetal tissue, as previously reported (16). However, unlike in CHI-D, NKX2.2 expression was not associated with a major disruption of the islet architecture or with an increase in the frequency of islet cell nucleomegaly (14, 16). It seems unlikely that enhanced/inappropriate insulin release is responsible for these observations because active CHI-A islets were found to have an appearance similar to that of control islets, and expressions of insulin, glucagon, somatostatin, and NKX2.2 were similar to those of controls.

The high levels of NXK2.2 expression in *δ*-cells and the clear parallels with NKX2.2/somatostatin coexpression in the fetal pancreas may imply an immature *δ*-cell function in CHI-A. Because endogenous somatostatin secretion in islets acts to inhibit insulin release, a naive population of *δ*-cells may have direct relevance to the disease profile. This is potentially attractive because it correlates with the clinical use of somatostatin analogues to restore the physiological inhibition of insulin release (1, 2). Furthermore, it also supports the notion that CHI could be part of a wider islet-based problem rather than a *β*-cell defect as previously suggested (27, 28). Similarly, the regional bias of the islet pathology to a particular lobe of the tissue and the appearance of mosaicism may support a developmental abnormality.

Despite an extensive number of transcription factors being involved in cell lineage determination from pancreatic stem cells and then endocrine progenitor cells, islet cells also retain an inherent plasticity in their developmental capacity and can undergo dedifferentiation. For example, the selective destruction of *β*-cells in rodents has been associated with *β*-cell regeneration following conversion of pancreatic *α*-cells (29) or *δ*-cells (30). This raises the further possibility that somatostatin/NKX2.2^+^ cell populations may represent a progenitor or intermediate pool of cells in the CHI islet that is triggered in response to the disease-modifying environment of the CHI pancreas.

In summary, these studies add to our current understanding of CHI-A and further illustrate the importance of studying human tissue in the disease context. Atypical cases of CHI may represent up to 10% of all CHI patients undergoing subtotal pancreatectomy for medically unresponsive CHI. Our data support the observations of others that the CHI-A pancreas can exhibit regional diversity, and this raises the possibility that patients could be treated with partial rather than subtotal pancreatectomy (31). However, we also recognize that in the absence of genetic biomarkers of disease and functional imaging techniques to localize the abnormal domain, this remains a theoretical possibility.
